# Institutional Needs

**Published:** 1913-04-19

**Authors:** 


### Institutional Needs.

### THE "LAWSON TAIT" BEDSTEAD.

The "Lawson Tait" type of bedstead, referred to in the article on "Institutional Beds and Bedding" in the Special Number of The Hospital on April 5, is, of course, the bedstead solely manufactured by Whitefield's Bedsteads, Limited, Watery Lane, Bordesley, Birmingham, who are the proprietors of the name "Lawson Tait," which is their registered trade-mark. It says much for the value of a trade-mark when it can be referred to as a standard in this way, but the actual bedstead can, of course, be obtained only from the above firm or their agents. Their London address is 10 Dane Street, High Holborn, W.C.

### THE "PHOSPHOCOSE" NERVE TONIC.

The Bayer Co., Ltd., 19 St. Dunstan's Hill, London, E.C., have placed this compound on the market. It consists of a pleasantly flavoured solution of Somatose containing 5 per cent. of sodium glycerophosphate. It is claimed that it possesses the value of their preparation known as "Somatose" as a tonic and restorative, the action of which is augmented by the addition of the glycerophosphate. The preparation is chiefly designed for the treatment of chronic nervous disorders.

### COFFEE WITHOUT CAFFEINE.

The Lifebelt Coffee Co., Ltd., has recently introduced a specially prepared coffee under the trade-mark "Lifebelt," which, in accordance with the evidence published by the company, is practically freed of the alkaloidal constituent known as caffeine, to which they maintain may be traced the harmful results of coffee drinking. The company claims that the aroma and other characteristics of ordinary coffee are fully retained by their preparation, and they especially recommend its use by those suffering from heart disease and nervous disorders who are unable to tolerate ordinary coffee. Coffee without caffeine may sound at first rather like beer without alcohol, but the "Lifebelt" is certainly a very pleasant drink.

### HYDROPYRIN AND KALMOPYRIN.

Messrs. Alfred Bishop, Ltd., are introducing two salts of acetyl-salicylic acid—viz., lithium acetyl-salicylate and calcium acetyl-salicylate—under the registered trade names Hydropyrin and Kalmopyrin. These products, it is stated, are readily and freely soluble in water, and for this, amongst other reasons, it is claimed that they are superior to all other forms of salicylates, for they can be prescribed in simple aqueous solutions, etc., thus obviating the necessity for administering tablets, and ensuring the immediate and full activity of the drugs.

